# Clinical validation of serum endocan (ESM-1) as a potential biomarker in patients with renal cell carcinoma

**DOI:** 10.18632/oncotarget.23087

**Published:** 2017-12-10

**Authors:** Kwang Hyun Kim, Hyung Ho Lee, Young Eun Yoon, Joon Chae Na, Sook Young Kim, Young In Cho, Sung Joon Hong, Woong Kyu Han

**Affiliations:** ^1^ Department of Urology, Ewha Womans University College of Medicine, Seoul, Republic of Korea; ^2^ Department of Medicine, The Graduate School of Yonsei University, Seoul, Republic of Korea; ^3^ Department of Urology, National Health Insurance Service Ilsan Hospital, Goyang, Republic of Korea; ^4^ Department of Urology, Hanyang University College of Medicine, Seoul, Republic of Korea; ^5^ Department of Urology, Urological Science Institute, Yonsei University College of Medicine, Seoul, Republic of Korea; ^6^ Brain Korean 21 PLUS Project for Medical Science, Yonsei University College of Medicine, Seoul, Republic of Korea

**Keywords:** carcinoma, renal cell, area under curve, early diagnosis

## Abstract

To determine the suitability of serum endocan (ESM-1) levels for diagnosing and monitoring renal cell carcinoma (RCC), we measure serum ESM-1 levels in 56 RCC patients who had undergone radical or partial nephrectomies and 56 age- and sex-matched healthy kidney donors. Measurements were made before and 1 month and 3 months after surgery. The areas under the curve (AUCs) were determined from receiver operating characteristic (ROC) analyses. RCC patients had higher mean serum ESM-1 levels than control subjects (0.59 ± 0.07 vs. 0.52 ± 0.08 ng/mL, *P* < 0.001), with an AUC of 0.721 (95% CI: 0.628–0.817). In patients with tumors larger than 2 cm (*n* = 40) and those with clear-cell histology (*n* = 44), the AUCs for ESM-1 were 0.771 and 0.721, respectively. In control subjects, serum ESM-1 levels were higher in older (>50 years) individuals (*P* < 0.001). Among the study cohort, the AUCs for ESM-1 were 0.813 in individuals 50 years of age or younger (*n* = 55) and 0.637 in individuals older than 50 years (*n* = 57). In RCC patients, serum ESM-1 levels were reduced 1 month (*P* = 0.047) and 3 months (*P* = 0.009) after surgery. These results suggest serum ESM-1 can serve as a serologic biomarker for diagnosing and monitoring RCC, particularly in patients younger than 50 years.

## INTRODUCTION

Renal cell carcinoma (RCC) is the most common malignant tumor in the kidney [1]. Although most RCC cases are diagnosed at an early stage, approximately 40% of RCC patients have locally advanced or metastatic disease [2]. The early detection of RCC is critical, because the cancer-specific survival rate drops from 66%–96% for patients with localized RCC to only 12%–36% for patients with locally advanced disease [3]. For patients with early detected small renal masses, treatment via a nephron sparing technique decreases the risk of chronic kidney disease and cardiovascular events [4, 5]. Thus, the identification of a biomarker for early stage disease ensures favorable patient outcomes.

Endocan, or endothelial cell-specific molecule-1 (ESM-1), is a soluble 50-kDa dermatan sulfate proteoglycan expressed by the vascular endothelium [6]. ESM-1 is overexpressed in obesity, during sepsis, and under inflammatory conditions, as well as in malignant tumors [7], and ESM-1 is associated with cardiovascular disease [8, 9]. In RCC tumors, which are typically hypervascular, ESM-1 is upregulated by angiogenic factors, such as vascular endothelial growth factor (VEGF) and platelet-derived growth factor (PDGF) [10]. A transcriptional profile of RCC revealed that ESM-1 is overexpressed in tissue samples of RCC [11]. The results from a study of a small number of patients by Leroy *et al.* [12] suggested that serum ESM-1 levels might serve as biomarkers in RCC. In this study, we prospectively enrolled patients with and without RCC and measured serum levels of ESM-1 before and after surgery to evaluate the potential of ESM-1 levels in diagnosing and monitoring RCC.

## RESULTS

Patient characteristics are summarized in Table 1. Of the 56 patients with RCC, 43 (76.8%) had T1a disease and 44 (78.6%) had clear-cell RCC. Although the control group was matched according to age and sex, patients in the RCC group were significantly older than those in the control group (*P* = 0.027). In the control group, the mean serum ESM-1 level did not vary according to sex (0.53 ± 0.08 ng/mL in male patients vs. 0.52 ± 0.77 ng/mL in female patients, *P* = 0.764) but was higher in older (>50 years) participants than in participants who were younger (≤50 years) (0.56 ± 0.06 ng/mL vs. 0.49 ± 0.08 ng/mL, *P* = 0.001). In the RCC group, the mean serum ESM-1 levels were not different between patients with clear-cell RCC and non–clear-cell RCC. In patients with clear-cell RCC, the serum ESM-1 levels were significantly higher in patients with large tumors and higher stages. However, no statistically significant differences were seen among patients in the entire RCC group (Table 2).

**Table 1:** Characteristics of study cohort

	RCC (*n* = 56)	Control (*n* = 56)	*P* value
Age (year)			
mean ± SD	52.9 ± 12.6	48.2 ± 9.0	0.027
median (IQR)	54 (44–62)	49 (40–57)	
Gender, *n* (%)			1.000
male	35 (62.5)	35 (62.5)	
female	21 (37.5)	21 (37.5)	
Size (cm)			
mean ± SD	3.64 ± 3.36		
median (IQR)	2.7 (2–3.8)		
Stage, *n* (%)			
T1a	43 (76.8)		
T1b	6 (10.7)		
T2a	2 (3.6)		
T2b	2 (3.6)		
T3a	3 (5.4)		
Furmann grade, *n* (%)			
I–II	36 (64.3)		
III–IV	20 (35.7)		
Cell type, *n* (%)			
clear cell	44 (78.6)		
non-clear	12 (21.4)		

RCC = renal cell cancer, SD = standard deviation, IQR = interquartile range.

**Table 2:** Serum endocan level according to tumor characteristics in patients with RCC

	serum endocan level	*P* value	serum endocan level (clear cell RCC only)	*P* value
Tumor size (cm)		0.095		0.006
≤2 (*n* = 16)	0.56 ± 0.08		0.54 ± 0.08	
>2, ≤4 (*n* = 27)	0.59 ± 0.06		0.59 ± 0.06	
>4 (*n* = 13)	0.61 ± 0.05		0.63 ± 0.02	
Stage		0.625		0.024
T1 (*n* = 49)	0.59 ± 0.07		0.58 ± 0.07	
>T2 (*n* = 7)	0.59 ± 0.07		0.63 ± 0.02	
Furmann grade		0.836		0.937
I–II (*n* = 36)	0.59 ± 0.06		0.59 ± 0.06	
III–IV (*n* = 20)	0.58 ± 0.08		0.59 ± 0.07	
Cell type		0.904		
clear cell (*n* = 44)	0.59 ± 0.06			
non-clear (*n* = 12)	0.59 ± 0.08			

**Figure 1:**
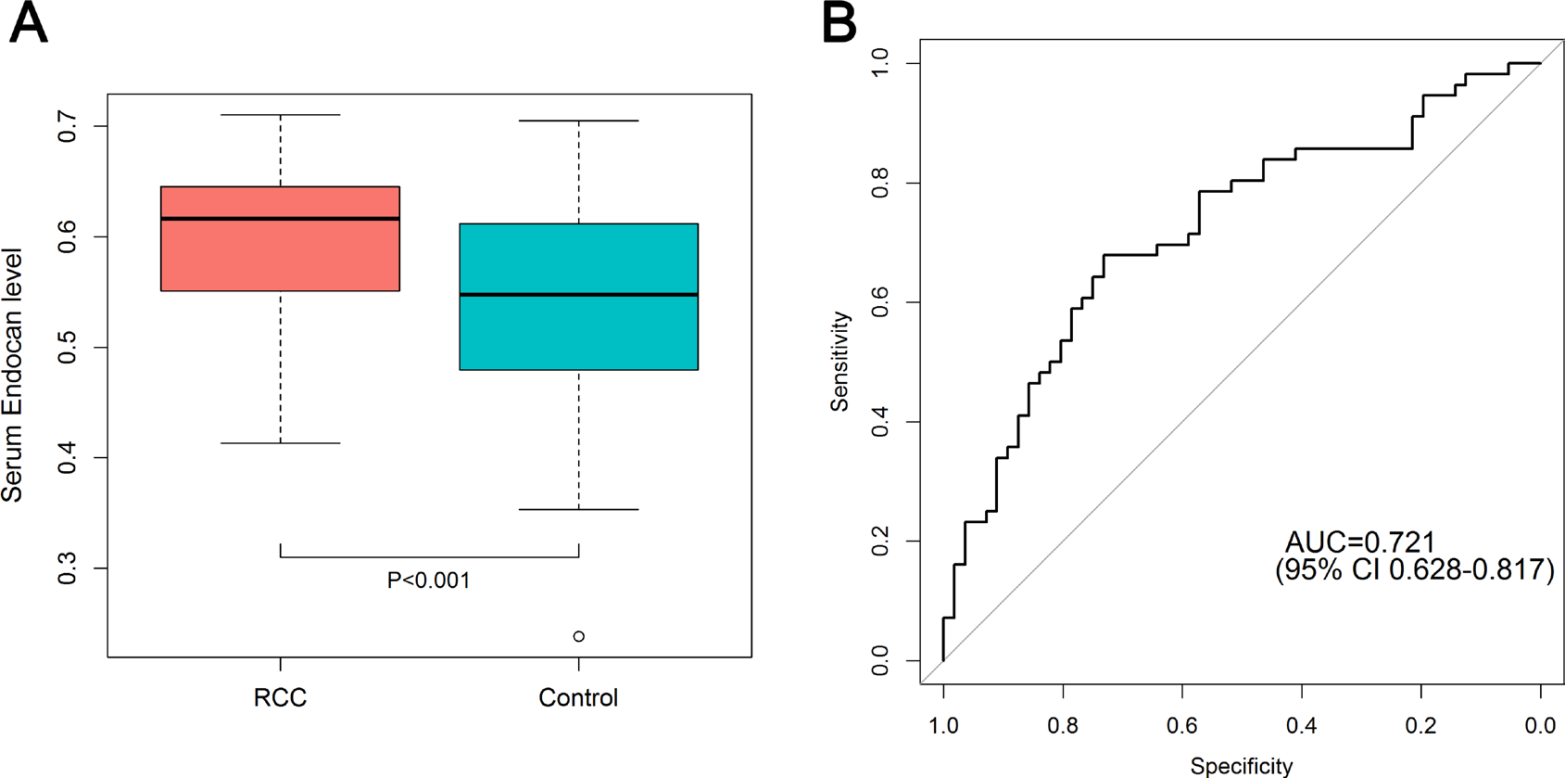
(**A**) Difference in serum ESM-1 level between RCC patients and control subjects. (**B**) ROC curve for serum ESM-1 comparing RCC patients (*n* = 56) and control subjects (*n* = 56). The AUC for serum ESM-1 was 0.721 (95% CI, 0.628–0.817).

**Figure 2:**
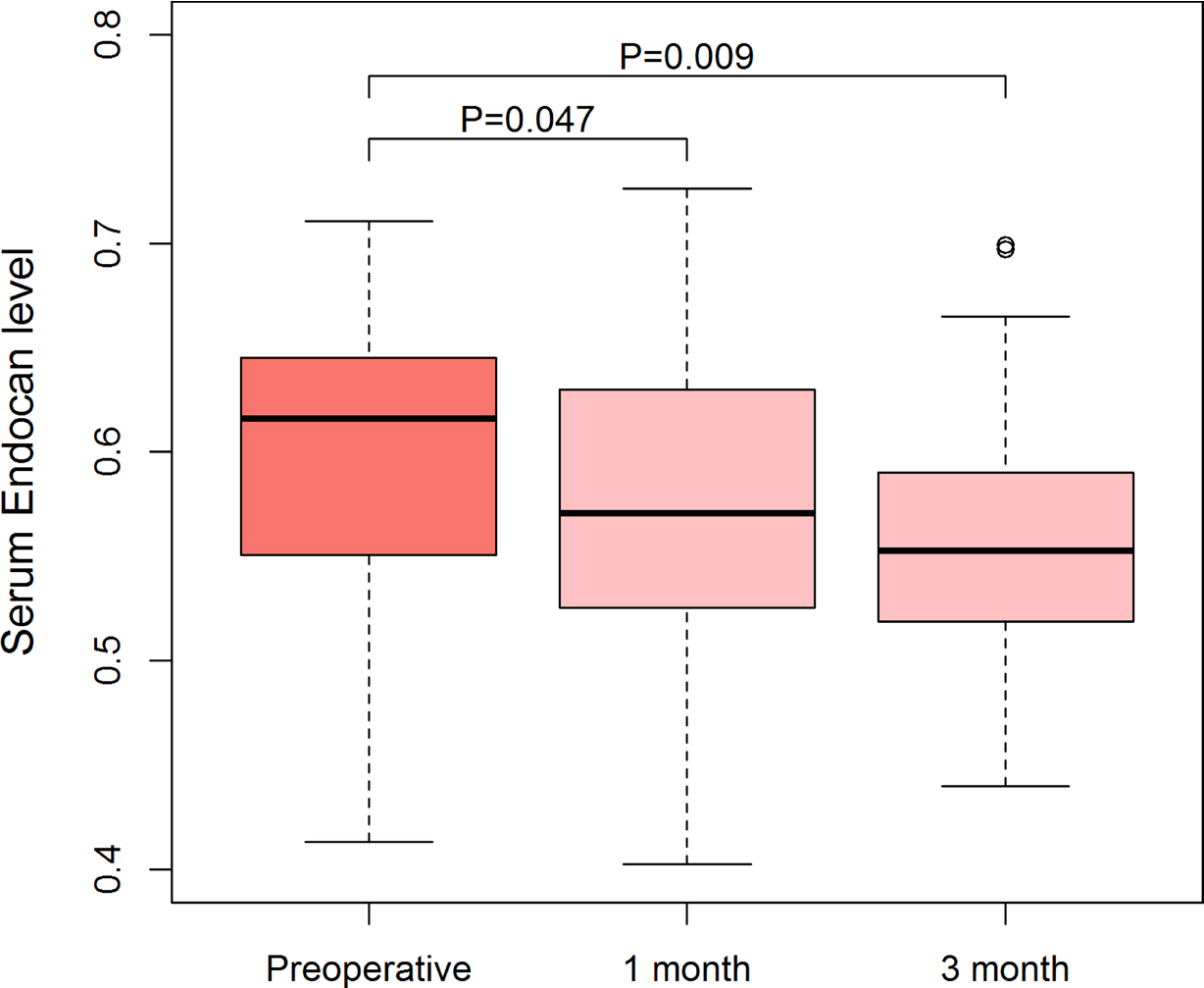
Serial changes in serum ESM-1 levels after surgery in patients with RCC Serum ESM-1 level declined after surgery, and preoperative and postoperative serum ESM-1 levels were compared by use of a paired *t*-test.

Patients with RCC had higher serum ESM-1 levels than control group participants (0.59 ± 0.07 mg/mL vs. 0.52 ± 0.08 ng/mL, *P* < 0.001) (Figure 1A). The area under the curve (AUC) of receiver operating characteristic (ROC) for ESM-1 level was 0.721 (95% CI: 0.628–0.817) (Figure 1B). With a cutoff value of 0.568 ng/mL (calculated by the Youden index), the sensitivity, specificity, positive-predictive values, and negative-predictive values were 67.9%, 73.2%, 30.5%, and 28.3%, respectively. In a subgroup analysis that included patients with tumor size larger than 2 cm (*n* = 40) and with clear-cell histology (*n* = 44), AUCs for ESM-1 levels were 0.771 and 0.721, respectively. Because of the older age of patients in the RCC group and the higher levels of serum ESM-1 in older participants, we performed a multivariate linear regression analysis. After controlling for effects of age and gender, we found that RCC significantly affects the serum level of ESM-1 (*P* < 0.001) (Table 3).When we divided the entire cohort according to age, the AUCs for ESM-1 were 0.813 in the younger group (age ≤ 50 years) and 0.637 in the older group (age > 50 years). When we analyzed the serial change in serum ESM-1 levels, the mean ESM-1 levels at 1 month and 3 months after surgery were 0.56 ± 0.07 ng/mL and 0.56 ± 0.06 ng/mL, respectively (Figure 2). In paired *t* tests, the serum ESM-1 levels at 1 month and 3 months after surgery were significantly lower than the preoperative serum ESM-1 level (*P* = 0.047 and *P* = 0.009, respectively).

**Table 3:** Multivariate linear regression analysis to evaluate whether the presence of RCC independently affects the level of serum ESM-1

	Univariate		Multivariate	
	β	*P*-value	β	*P*-value
Age	0.239	0.011	0.165	0.070
Gender	–0.050	0.600	–0.035	0.693
RCC (vs control)	0.375	<0.001	0.340	<0.001

## DISCUSSION

In this study, we found that serum ESM-1 levels were higher in patients with RCC than in healthy individuals. Although serum ESM-1 levels were influenced by age, they were also promoted by the presence of RCC. Thus, serum ESM-1 can potentially be used as a diagnostic marker for RCC.

Increased levels of ESM-1 have been found in the sera of patients with colorectal cancer, hepatocellular carcinoma, and acute leukemia [13–15]. Moreover, immunohistochemical studies have shown that elevated ESM-1 expression correlates with unfavorable prognoses in different types of cancers, including glioblastoma, colon cancer, and hepatocellular carcinoma, as well as RCC [12, 16–18]. Because angiogenesis is an important process in tumor progression, factors that are influenced by or related to this process can be useful for diagnostic and prognostic determinations. Tumor cells in RCC are characterized by an increased stability of hypoxia-inducible factor (HIF) and the subsequent induction of VEGF expression [19]. Rennel *et al.* [10] demonstrated that ESM-1 is secreted from endothelial cells in response to VEGF, suggesting ESM-1 as potential tumor marker of RCC. Subsequent study has shown that serum ESM-1 levels increased in patients with RCC but not in healthy control subjects [12]. However, the study focused on overexpression of ESM-1 in clear cell RCC, and the small number of patients were included in the ELISA analysis. Moreover, the previous study found that ESM-1 levels were increased in clear cell RCC but not in papillary RCC, and ESM-1 immunoreactivity was very rare in papillary RCC cells. However, in our study, serum ESM-1 levels did not differ between patients with clear cell and non–clear cell RCC. Although activation of HIF and VEGF have been described mostly in clear cell RCC, HIF activity in other subtypes of RCC has been suggested [20]. Moreover, in our study, the diagnostic performance of ESM-1 was not improved in the clear cell RCC subgroup. Thus, we speculate that serum ESM-1 might serve as a more generalized biomarker and is not restricted to clear cell RCC.

In the results of the subgroup analysis, we found that the diagnostic performance of serum ESM-1 for RCC improved in patients with tumor size larger than 2 cm. It is attributable to the reflection of the tumor burden by serum ESM-1, which is in line with the previous study [12]. With the tumor size, the diagnostic performance was greatly improved in the young cohort, which included individuals aged 50 years or younger. The diagnostic performance of serum ESM-1 is notable in the young cohort. We found that the level of serum ESM-1 increased with age. The aging process is closely related to chronic inflammation, which also influences the expression of ESM-1. Therefore, although ESM-1 levels were increased in sera from patients with RCC independent of age, RCC might be more difficult to diagnose in elderly patients by ESM-1 levels alone. However, evidence has shown that small renal masses, which are typically slow-growing nonaggressive tumors, might not be a factor in the survival of elderly patients [21]. Thus, ESM-1 levels in serum might be more useful for diagnosing RCC in younger patients.

The measurement of serum ESM-1 levels after the surgical removal of RCC was not performed in previous studies. We found that the levels decreased postoperatively, although they remained higher than in the control group. We could not exclude an effect from the surgery itself, because postoperative serum ESM-1 levels were not obtained from the healthy control subjects. Nevertheless, the differences were statistically significant in paired *t*-tests. The decreased levels were maintained for up to 3 months postoperatively. We believe these findings suggests that serum ESM-1 can be a surveillance marker for RCC.

Although elevated serum levels of ESM-1 in RCC have been reported, this study included the largest number of patients with RCC and healthy control subjects. Our results indicated that ESM-1 can be used as both a diagnostic and a surveillance biomarker. In addition, we found that ESM-1 showed improved diagnostic performance in a young cohort. These novel findings represent strengths of our study.

Some limitations of this study must be considered: First, this study did not include patients with benign renal masses. Currently, we only have a small number of patients with benign renal masses in our database, and a larger cohort is needed to investigate the efficacy of serum ESM-1 levels in differentiating RCC from benign renal masses. Second, serum ESM-1 levels are also elevated in other types of cancers and thus are not specific for RCC. However, unlike hepatocellular carcinoma and colorectal cancer, RCC has no serologic biomarker for diagnosis. Third, the prognostic ability of ESM-1 in RCC was not assessed in this study, because only two cases of recurrence were seen during the median 27 months of follow-up. Therefore, a longer follow-up involving patients with more advanced stages of RCC is required to evaluate the use of serum ESM-1 levels for prognosis.

The results of our study show that serum ESM-1 levels can serve as a diagnostic biomarker for RCC, particularly in patients younger than 50 years. Moreover, the assessment of serum ESM-1 levels can be utilized for monitoring patients with RCC after surgery. Although a validation in a larger cohort is necessary, we conclude that serum ESM-1 can potentially be clinically applied for diagnosing and monitoring RCC.

## MATERIALS AND METHODS

### Study participants and blood sampling

From October 2013 to March 2015, we prospectively enrolled patients undergoing nephrectomies in the urology department at Severance Hospital. In total, 56 patients with pathologically confirmed RCC underwent partial or radical nephrectomies. For the control group, we included 56 age-matched and sex-matched individuals from a cohort of healthy kidney donors who had undergone donor nephrectomies during the same period. This study was approved by the Institutional Review Board of Severance Hospital (IRB no. 4-2013-0166), and informed consent was obtained from all participants before study enrollment.

Blood sampling was performed the day before surgery for all participants and at 1 month and 3 months after surgery for patients in the RCC group. Blood samples were centrifuged for 15 minutes at 1,000 × *g* within 30 minutes of collection, and the sera were divided into aliquots and stored at −80°C for subsequent measurements of ESM-1 levels.

### Assessment of ESM-1 levels

In all serum samples, the level of ESM-1 was quantified by use of a commercially available ELISA kit (Lunginnov s.a.s., Lille, France). Serum was diluted twofold before analysis. Experiments were performed according to manufacturer’s instructions. Microwell plates were coated with 100 μL of capture antibody (2 μg/mL) and incubated overnight at 4°C. The coated plates were blocked with 5% skim milk in tris buffered saline (TBS) for 1 hour at 37°C. After a washing step with phosphate buffered saline (PBS), the plates were incubated with 100 μL of serum specimen for 1 hour, and then washed and incubated with 100 μL of secondary antibody (diluted 1:10,000) for 1 hour at room temperature. After the washing, 100 μL of streptavidin-horseradish peroxidase (1:10,000) was added and incubated for 30 minutes. After a final washing, 50 μL of tetramethylbenzidine substrate was added and the reaction was stopped with 1 N H_2_SO_4._ The absorbance was determined at 450 nm on a spectrophotometer. All assays were performed in duplicate.

### Statistical analysis

ESM-1 levels in the control group were compared according to the sex and age (≤50 years vs. >50 years) of the participants. Serum ESM-1 levels were then compared between control subjects and RCC patients, as well as according to tumor size, pathologic stage, Fuhrman grade, and histology in RCC patients. ROC curves were generated and the AUCs were calculated. The optimal cutoff value, which maximizes sensitivity and specificity, was obtained by use of the Youden index. In addition, we performed subgroup analyses of RCC patients with tumor sizes larger than 2 cm or with clear-cell histology.

Although RCC and control groups were matched by age and sex, patients in the RCC group were older than those in the control group. Because of the higher levels of serum ESM-1 in older participants than in their counterparts in the control group, we performed multivariate linear regression analyses to evaluate whether the presence of RCC significantly increases the level of serum ESM-1.

Preoperative and postoperative (at 1 month and 3 months) ESM-1 levels were compared. The quantitative values were compared by use of Student’s *t*-tests or one-way analyses of variance, whereas qualitative variables were compared by use of chi-square or Fisher’s exact tests. Statistical analyses were performed by the Statistical Package for Social Science for Windows, Version 18.0 (SPSS, Chicago, IL, USA). A *P*-value < 0.05 was considered significant, and all *P*-values were two-sided. The ROC analyses and plotting graphics were performed by R Version 3.2.5 software (http://www.r-project.org).
